# From Printability to Biofunctionality: 3D-Printed Hydrogel Scaffolds for Multi-Tissue Engineering

**DOI:** 10.3390/gels12070585

**Published:** 2026-07-02

**Authors:** Yufei Zhang, Chenyu Shen, Yuxin Liu, Jinfeng Zhang, Zhangkang Li

**Affiliations:** 1 Department of Medical Imaging, Suzhou Medical College, Soochow University, Suzhou 215021, China; 2 Basic Medical Research Center, Medical School, Nantong University, Nantong 226001, China; 3 Department of Chemical and Petroleum Engineering, University of Calgary, Calgary, AB T2N 1N4, Canada; yuxin.liu3@ucalgary.ca; 4 Department of Biomedical Engineering, University of Calgary, Calgary, AB T2N 1N4, Canada

**Keywords:** 3D printing, hydrogel scaffolds, bioinks, biofunctionality, tissue engineering, regenerative medicine

## Abstract

3D-printed hydrogel scaffolds have emerged as important platforms in tissue engineering and regenerative medicine owing to their extracellular matrix-like three-dimensional hydrated networks, tunable physicochemical properties, and ability to spatially organize cells, bioactive factors, and scaffold architectures. Early studies mainly focused on the printability, shape fidelity, and biocompatibility of hydrogel inks, whereas current research has gradually shifted toward the construction of bioactive scaffolds with tissue-specific functions. Because different tissues exhibit distinct requirements in terms of mechanical properties, cellular microenvironment, vascularization, innervation, degradation behavior, and functional maturation, the design of 3D-printed hydrogel scaffolds should comprehensively consider material composition, printing strategy, biofactor delivery, and tissue-specific functional demands. In this review, we focus on the transition from printability to biofunctionality and systematically summarize recent advances in 3D-printed hydrogel scaffolds for multi-tissue engineering. Particular emphasis is placed on regenerative applications of 3D-printed hydrogel scaffolds in bone, cartilage, vascular, neural, and skin tissue engineering. Finally, we discuss the major challenges associated with 3D-printed hydrogel scaffolds and further highlight future directions. This review aims to provide a systematic reference for the functional design and application of 3D-printed hydrogel scaffolds in multi-tissue engineering.

## 1. Introduction

Hydrogels are three-dimensional hydrophilic polymer networks characterized by high water content, tunable physicochemical properties, and structural similarity to the natural extracellular matrix [1,2,3,4,5]. Owing to these features, hydrogels have been widely applied in tissue engineering and regenerative medicine [6,7,8,9,10,11,12]. In tissue engineering, hydrogels are often engineered into scaffolds because they can serve as temporary extracellular matrix-like frameworks to support tissue regeneration. Similar to the supporting framework required during building construction, hydrogel scaffolds provide a provisional three-dimensional template that helps maintain defect space, support cell attachment and growth, and guide the formation of new tissue. Therefore, hydrogel scaffolds are not merely passive fillers, but functional matrices that provide cells with a hydrated three-dimensional microenvironment for adhesion, proliferation, migration, differentiation, and extracellular matrix deposition [13,14]. Compared with conventional two-dimensional culture systems or dense biomaterial implants, hydrogel scaffolds can better mimic the hydrated and porous microenvironment of native tissues. Their porous structures facilitate the transport of nutrients, oxygen, metabolic waste, and bioactive molecules, which is essential for cell survival and tissue ingrowth. In addition, the mechanical properties, degradation behavior, biochemical cues, and biological functions of hydrogels can be regulated through polymer selection, chemical modification, crosslinking strategies, and the incorporation of cells or bioactive factors. These characteristics make hydrogel scaffolds attractive platforms for the repair and regeneration of various tissues, including bone, cartilage, skin, neural tissue, cardiac tissue, vascular tissue, and complex organ models [15,16].

However, native tissues usually possess complex and highly organized architectures, such as interconnected pores, anisotropic fiber arrangements, vascular-like networks, gradient interfaces, and region-specific cellular distributions. Traditional hydrogel scaffold fabrication methods, including casting and molding, as well as post-processing techniques such as freeze-drying, and bottom-up self-assembly strategies, often have limited ability to precisely control scaffold geometry, internal pore architecture, spatial heterogeneity, and cell distribution. In particular, casting and molding mainly define the macroscopic geometry of scaffolds, whereas freeze-drying relies on ice crystal templating during solvent sublimation, typically resulting in randomly distributed and non-programmable porous structures. In contrast, self-assembly is driven by molecular or supramolecular interactions and mainly occurs at the network or microscale level, which provides limited direct control over macroscopic scaffold architecture. As a result, conventional hydrogel scaffolds may fail to fully recapitulate the structural complexity and functional requirements of native tissues. Three-dimensional printing provides an effective strategy to overcome these limitations. Through layer-by-layer fabrication, 3D printing enables the construction of hydrogel scaffolds with predefined shapes, controllable internal architectures, and programmable spatial organization of cells and bioactive components [17,18]. By adjusting printing parameters and scaffold design, hydrogel scaffolds can be fabricated with specific pore size, porosity, filament orientation, interconnected channels, and gradient structures to meet the requirements of different tissues. Moreover, 3D printing can be combined with medical imaging data to fabricate patient-specific scaffolds and further supports the construction of multi-material and cell-laden tissue constructs.

With the development of printing technologies and hydrogel bioinks, 3D-printed hydrogel scaffolds have been increasingly applied in multiple tissue engineering fields. However, the goal of 3D-printed hydrogel scaffold engineering is no longer limited to improving printability or shape fidelity. Instead, increasing attention has shifted toward whether printed hydrogel scaffolds can provide tissue-specific biofunctionality and promote functional tissue regeneration. Different tissues have distinct requirements in terms of mechanical strength, degradation behavior, cellular microenvironment, vascularization, innervation, biochemical signaling, and functional maturation [19,20,21]. Therefore, the design of 3D-printed hydrogel scaffolds should integrate hydrogel composition, printing strategy, scaffold architecture, cell type, bioactive factor delivery, and tissue-specific functional demands. In this review, we first summarize the major printing methods used for hydrogel scaffold fabrication and discuss the key design considerations of printable hydrogel inks. We then focus on the applications of 3D-printed hydrogel scaffolds in different tissue engineering fields, including bone, cartilage, vascular, neural, and skin tissue engineering. Finally, we discuss the current limitations and future directions of 3D-printed hydrogel scaffolds, with particular emphasis on the transition from printability to tissue-specific biofunctionality.

## 2. Printing Methods

**Figure 1:**
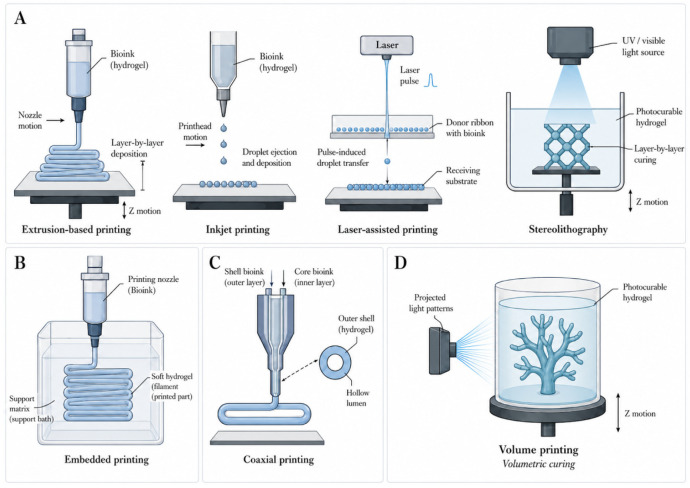
(**A**) Schematic illustration of commonly used methods for printing hydrogel scaffolds. (**B**) Embedded printing strategy for hydrogel scaffold fabrication. (**C**) Coaxial extrusion-based printing method for hydrogel scaffold fabrication. (**D**) Volumetric printing approach for hydrogel scaffold fabrication.

At present, various three-dimensional printing technologies have been applied to fabricate hydrogel scaffolds, mainly including extrusion-based printing, inkjet printing, laser-assisted printing and stereolithography (Figure 1A) [22,23,24]. These printing methods differ in material compatibility, printing resolution, fabrication speed, cell compatibility, and structural complexity. Therefore, the selection of an appropriate printing strategy should be guided by the rheological properties and crosslinking mechanisms of hydrogel inks, as well as the functional requirements of the target tissue.

Extrusion-based printing is one of the most widely used methods for hydrogel scaffold fabrication. In this approach, hydrogel inks are continuously extruded through a nozzle driven by pneumatic pressure, piston force, or screw-based mechanisms, and are deposited layer by layer along a predefined printing path to form three-dimensional structures [25,26]. Extrusion-based printing is compatible with a broad range of hydrogel viscosities, especially bioinks with viscoelastic and shear-thinning properties, and it allows cell-laden printing under relatively mild conditions. Therefore, this method has been widely used to construct hydrogel scaffolds for bone, cartilage, skin, vascular, and cardiac tissue engineering. However, the shear stress generated during extrusion may affect cell viability, and its printing resolution is generally lower than that of photopolymerization-based printing methods. Inkjet printing deposits low-viscosity hydrogel inks as microdroplets onto target regions through thermal, piezoelectric, or acoustic actuation. This method offers several advantages, including high printing speed, low material consumption, and the ability to spatially pattern cells and bioactive factors. It is particularly suitable for constructing cell arrays, drug screening platforms, and localized biofactor delivery systems. However, inkjet printing has strict requirements for ink viscosity, surface tension, and cell concentration, and is also limited by nozzle clogging and droplet stability [27,28]. Laser-assisted printing uses laser energy to induce the transfer or deposition of bioinks, enabling high-precision patterning of cells and biomaterials without the need for a nozzle. Because this nozzle-free process avoids nozzle clogging and allows high-resolution deposition, it is suitable for fabricating precise tissue-like structures. Nevertheless, the high cost of equipment, operational complexity, and potential effects of laser energy on cell viability still limit its broader application [29,30]. Photopolymerization-based printing methods, such as stereolithography and digital light processing, are mainly used for photocrosslinkable hydrogel systems containing photosensitive functional groups. These techniques induce rapid gelation of hydrogel precursors through light exposure and offer high printing resolution, fast fabrication speed, and excellent structural accuracy. Since many hydrogel systems can be rendered photocrosslinkable through methacrylation, acrylation, or other photosensitive modifications, photopolymerization-based printing is particularly suitable for fabricating high-resolution hydrogel scaffolds, microstructured scaffolds, and complex tissue models [31,32]. However, the design of photocrosslinkable hydrogel inks requires careful consideration of photosensitive groups, photoinitiator biocompatibility, light wavelength, light intensity, exposure time, curing depth, gelation kinetics, and potential phototoxicity, in order to balance printing accuracy, structural stability, and cell viability.

Overall, no single printing method is universally superior; rather, each approach is suitable for specific hydrogel systems and tissue engineering requirements. Among them, extrusion-based printing and photopolymerization-based printing are currently the most mainstream and representative strategies for hydrogel scaffold fabrication. Extrusion-based printing offers broad material compatibility, strong cell-loading capacity, and great potential for multi-material fabrication, and it can be further extended into embedded printing, coaxial printing, and multi-material printing for constructing complex soft tissue structures, tubular architectures, and gradient scaffolds [33]. Embedded printing generally deposits hydrogel inks within a supportive gel bath or granular bath, allowing low-viscosity or mechanically weak hydrogels to maintain complex shapes before crosslinking (Figure 1B). Therefore, it is suitable for fabricating freeform architectures, soft tissue models, and complex vascular networks [34,35]. Coaxial printing uses a coaxial nozzle to simultaneously extrude core and shell materials, enabling the formation of hollow tubular structures, core–shell architectures, or delivery-functional composite scaffolds (Figure 1C). It is particularly valuable for constructing vascular structures, neural conduits, and controlled release systems [36,37]. In contrast, photopolymerization-based printing offers clear advantages in printing resolution, structural accuracy, and the fabrication of complex microarchitectures. It relies on different light sources, such as UV and visible light, to selectively cure photocurable hydrogel precursors, enabling precise layer-by-layer construction of micro- and macro-scale structures. This method allows for rapid fabrication of intricate geometries, fine features, and overhanging architectures that are difficult to achieve with extrusion-based techniques. The most representative method is stereolithography (SLA). Inspired by SLA, volume printing has gradually been developed as a new approach (Figure 1D). Volume printing, a recently developed variant, differs from traditional layer-by-layer approaches by curing the entire 3D structure simultaneously within a photocurable resin using volumetric light patterns [38,39]. This enables much faster fabrication times and smooth continuous structures, making it particularly suitable for producing complex hydrogel scaffolds in tissue engineering applications. A wide range of 3D printing techniques has been developed, and each technique is inherently associated with specific requirements for hydrogel ink properties. Accordingly, the matching between printing modality and ink formulation plays a central role in determining printability, cytocompatibility, and mechanical integrity.

## 3. Hydrogel Bioink Design

The design of hydrogel bioinks is critical for successful 3D printing and subsequent tissue regeneration. Different printing modalities impose distinct requirements on bioink properties. Extrusion-based printing requires hydrogel bioinks to have tunable rheological properties to ensure continuous filament extrusion and shape fidelity. The bioink should exhibit shear-thinning behavior, allowing it to flow smoothly during extrusion and rapidly recover viscosity after deposition to maintain structural stability [40]. Viscosity needs to be balanced: too low and the filament collapses, too high and it cannot be extruded. Additionally, the bioink must be compatible with cell encapsulation, maintaining high cell viability under shear stress and providing sufficient mechanical support to preserve the printed scaffold architecture during culture. Photopolymerization-based printing, such as SLA, requires bioinks to be photocurable and to contain functional groups with C=C double bonds to rapidly form crosslinked networks under light exposure. The bioink must balance printability, crosslinking rate, and cell viability [41,42]. Photoinitiators may exhibit cytotoxic effects, which should be carefully considered.

Bioinks are generally composed of polymers, which can be natural or synthetic. Natural polymers, such as gelatin, alginate, and collagen, offer excellent biocompatibility and cell-interactive motifs, promoting cell adhesion, proliferation, and differentiation, but often exhibit weak mechanical properties and batch-to-batch variability [43,44]. Synthetic polymers, such as PEG derivatives or PVA, provide tunable mechanical properties, stable material quality, and chemical functionalization, but usually require modification to support cell adhesion and bioactivity. Therefore, some natural polymers that have been chemically modified with double bonds are particularly suitable for scaffold printing [45,46]. For example, methacrylated gelatin (GelMA) combines cell compatibility with photocrosslinkable properties, making it highly suitable for photopolymerization-based 3D printing of hydrogel scaffolds [47]. Many hydrogel-based bioinks can be flexibly applied to different in vivo or tissue-engineering scenarios by modulating their crosslinking mechanisms, mechanical properties, degradation behavior, cell-loading strategies, and functional components. Therefore, the relationship between bioink composition and tissue application is often flexible rather than fixed. GelMA is currently one of the most widely used and promising hydrogel bioinks, owing to its excellent photocrosslinkability, high compatibility with light-assisted printing technologies, relatively high printing resolution, tunable mechanical properties, favorable cytocompatibility, and broad applicability in both 2D and 3D cell culture/bioprinting systems. These features make GelMA not merely suitable for a specific tissue type, but rather a flexible bioink platform that can be further combined with other materials or functional components and extended to different tissue and multi-tissue engineering applications.

Overall, the selection of hydrogel bioinks involves a trade-off between printability, mechanical properties, and biological functionality. For extrusion-based printing, rheological tuning and shear-thinning behavior are critical, whereas for photopolymerization-based printing, photocurable functionality and light-induced crosslinking kinetics are essential. Hybrid formulations combining natural and synthetic polymers often provide an optimal balance for tissue-specific scaffold design. Detailed summaries are provided in Table 1 and Table 2.

## 4. Osteochondral and Bone Repair

Articular cartilage and subchondral bone repair, particularly the repair of knee osteochondral defects, represents one of the important applications of 3D-printed hydrogel scaffolds [52,53]. Articular cartilage has limited self-healing capacity because it is avascular and contains only a low density of resident chondrocytes. Once cartilage damage extends into the subchondral bone, the repair process becomes more complex, as cartilage and bone possess distinct biological lineages, tissue structures, mechanical properties, and biochemical microenvironments [54,55]. Therefore, pure hydrogel scaffolds with a single composition are usually insufficient for osteochondral repair. On the one hand, pure hydrogels often exhibit limited mechanical strength and compressive resistance, making it difficult to withstand the compressive loading of the knee joint and maintain the defect space over time. On the other hand, homogeneous hydrogel scaffolds lack the spatial heterogeneity of native osteochondral tissue and therefore cannot simultaneously recapitulate the distinct regenerative microenvironments of the cartilage layer and the subchondral bone layer. For knee osteochondral defect repair, an ideal scaffold should not only provide a cartilage-like highly hydrated microenvironment, but also possess sufficient mechanical support, interconnected porous structures, and region-specific biological functions. The scaffold should support cell adhesion and migration, promote nutrient transport and tissue ingrowth, and provide chondrogenic and osteogenic cues for the cartilage layer and subchondral bone layer, respectively. Therefore, spatially biomimetic bilayer or multilayer scaffolds are more suitable for osteochondral defect repair than single homogeneous hydrogel scaffolds. In this context, 3D printing provides an effective strategy for optimizing both the structure and function of hydrogel scaffolds. Compared with conventional molding or casting methods, 3D printing enables precise control over scaffold geometry, pore architecture, filament alignment, mechanical gradients, and the spatial distribution of bioactive cues. More importantly, 3D printing allows the fabrication of bilayer or multilayer hydrogel scaffolds with region-specific compositions, in which the upper layer mimics the cartilage microenvironment and the lower layer mimics the subchondral bone microenvironment, thereby guiding chondrogenic and osteogenic regeneration, respectively. In addition, 3D printing enables the programmable distribution of cells, growth factors, or exosomes in different regions, providing spatial guidance for coordinated repair of the osteochondral unit. Therefore, 3D-printed hydrogel scaffolds for bone and osteochondral repair often integrate design strategies such as bilayer architectures, gradient stiffness, hierarchical porosity, reinforced networks, and bioactive factor delivery.

**Figure 2:**
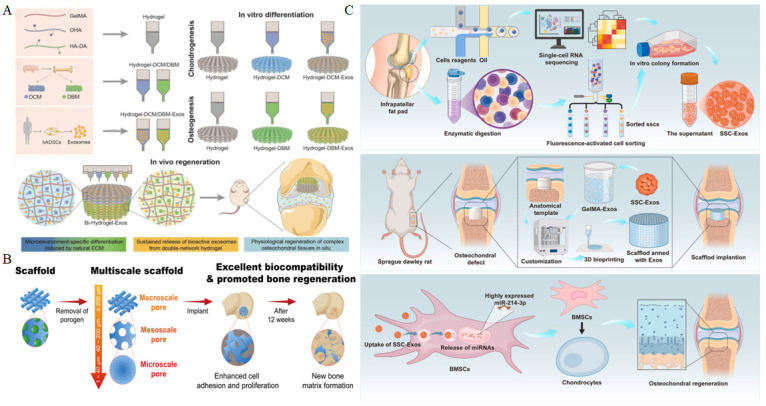
(**A**) 3D printing of microenvironment-specific biomimetic hydrogel scaffolds for the repairing of osteochondral defects [60] (copyright © 2023, Li et al. under the terms of the Creative Commons CC BY 4.0). (**B**) Promotion of bone defect repairs using multiscale 3D-printed silk porous hydrogel scaffolds [61] (adapted with permission from Liu et al. copyright 2025 Elsevier). (**C**) The construction of a biomimetic 3D-printed scaffold armed with SSC-Exos for osteochondral regeneration [62] (copyright © 2025, Lou et al. under the terms of the Creative Commons CC BY 4.0).

In addition to structural optimization, the incorporation of bioactive components is also an important strategy to enhance the regenerative performance of 3D-printed hydrogel scaffolds. Among these components, exosomes have been frequently incorporated into hydrogel scaffolds as cell-free bioactive regulators for bone and osteochondral repair. Exosomes are particularly attractive because they can mediate paracrine signaling by delivering bioactive molecules, including microRNAs, proteins, and lipids, to recipient cells [56,57,58,59]. These cargos can regulate cell proliferation, migration, osteogenic differentiation, chondrogenic differentiation, angiogenesis, and immune responses. Compared with direct cell transplantation, exosome-based strategies may reduce risks associated with cell survival, uncontrolled differentiation, and immune rejection, while still preserving important regenerative signals. Therefore, loading exosomes into 3D-printed hydrogel scaffolds can provide sustained and localized biological stimulation during tissue repair. For example, Li et al. developed a 3D-bioprinted exosome-reinforced bilayer hydrogel scaffold for cartilage and subchondral bone regeneration (Figure 2A) [60]. This spatially designed scaffold provided tissue-specific microenvironments through a dual-network system, resulting in enhanced natural extracellular matrix (ECM) reconstruction and cartilage-related collagen formation during tissue regeneration. In this design, methacrylated gelatin (GelMA) formed a relatively rigid and brittle first network after photocrosslinking, while Schiff base bonds contributed to a softer and tougher second network. This dual-network architecture enhanced the scaffold’s bioactivity, chondrogenic and osteogenic properties, and promoted simultaneous regeneration of cartilage and subchondral bone in a rat model of critical-sized osteochondral defects. In another study, Liu et al. reported multiscale 3D-printed silk hydrogel scaffolds for bone defect repair (Figure 2B) [61]. By incorporating dextran gel particles as sacrificial porogens, the silk hydrogel scaffolds achieved hierarchical pore structures favorable for cell adhesion, proliferation, and migration. Both in vitro and in vivo results showed that these scaffolds supported robust cellular activity and significantly enhanced bone regeneration compared with conventional scaffold designs. This work highlights the importance of multiscale porosity and structural optimization in improving the regenerative performance of hydrogel-based bone scaffolds. Similarly, Lou et al. fabricated a 3D-printed hydrogel scaffold loaded with skeletal stem cell-derived exosomes for osteochondral defect repair (Figure 2C) [62]. In a rat osteochondral defect model, the scaffold loaded with Skeletal Stem Cell (SSC)-derived exosomes exhibited excellent osteochondral regenerative effects and promoted synchronous repair of both cartilage and subchondral bone. In vitro experiments further demonstrated that SSC-derived exosomes significantly enhanced the chondrogenic differentiation of bone marrow mesenchymal stem cells. Mechanistically, the study identified the miR-214-3p/JAG2 axis as a key pathway involved in SSC-exosome-mediated cartilage regeneration. This strategy suggests that combining 3D-printed hydrogel scaffolds with tissue-specific exosomes may provide an effective and potentially translatable approach for osteochondral defect repair.

Overall, bone and osteochondral repair require 3D-printed hydrogel scaffolds with both structural and biological functions. For cartilage repair, the scaffold should provide a soft, highly hydrated, and chondrogenic microenvironment to support extracellular matrix deposition. For subchondral bone repair, the scaffold should provide stronger mechanical support, osteogenic cues, interconnected porous structures, and possibly pro-angiogenic regulation. Therefore, future hydrogel scaffolds for osteochondral regeneration should not be designed as single homogeneous materials, but rather as spatially organized, mechanically graded, and biologically functional three-dimensional constructs that can guide the coordinated regeneration of cartilage and subchondral bone. A clinically representative example is matrix-induced autologous chondrocyte implantation (MACI), an FDA-approved scaffold-based technique for cartilage defect repair. In MACI, autologous chondrocytes are first isolated from a small cartilage biopsy, expanded in vitro, seeded onto a porcine collagen membrane, and then implanted into the cartilage defect site. After implantation, the collagen membrane provides a temporary supportive matrix for the delivered chondrocytes, allowing them to survive, remain at the defect site, produce cartilage-related extracellular matrix, and contribute to defect repair. Although MACI is not a 3D-printed hydrogel scaffold and mainly targets cartilage rather than integrated osteochondral regeneration, its clinical use demonstrates the feasibility of a cell-scaffold combined strategy for cartilage repair. This provides a useful translational reference for future printable hydrogel scaffolds, particularly in terms of efficient cell delivery, scaffold biocompatibility, defect adaptation, surgical handling, stable fixation, and long-term safety.

## 5. Vascular Regeneration

As discussed above, articular cartilage has limited self-repair capacity partly because it lacks blood vessels. However, in many other tissue repair scenarios, especially bone regeneration and large-volume tissue reconstruction, vascularization is indispensable for successful tissue regeneration [63]. Blood vessels provide oxygen, nutrients, immune cells, and biochemical signals, while also removing metabolic waste from the defect site. In bone repair, vascular networks are particularly important because osteogenesis and angiogenesis are closely coupled processes [64]. Newly formed blood vessels not only maintain cell survival in the defect region, but also support the recruitment of osteoprogenitor cells, mineral deposition, and remodeling of newly formed bone. Without sufficient vascularization, the central region of large scaffolds may suffer from hypoxia, nutrient deprivation, and tissue necrosis, which severely limits the regeneration outcome [65]. More specifically, vascularization is not simply the formation of channel-like structures within scaffolds, but involves multiple processes, including host vessel ingrowth into the scaffold, endothelial cell migration and alignment, microvascular network formation, establishment of blood perfusion, and integration of newly formed vessels with the host vascular system. In the early stage of tissue repair, local hypoxia and the inflammatory microenvironment can induce the release of pro-angiogenic factors, such as vascular endothelial growth factor, fibroblast growth factor, and platelet-derived growth factor, thereby promoting endothelial cell migration, proliferation, and the formation of lumen-like structures. Subsequently, the newly formed vascular structures need to be gradually stabilized through extracellular matrix remodeling and the involvement of supporting cells, such as pericytes and vascular smooth muscle cells, to maintain vascular integrity and long-term perfusion function.

**Figure 3:**
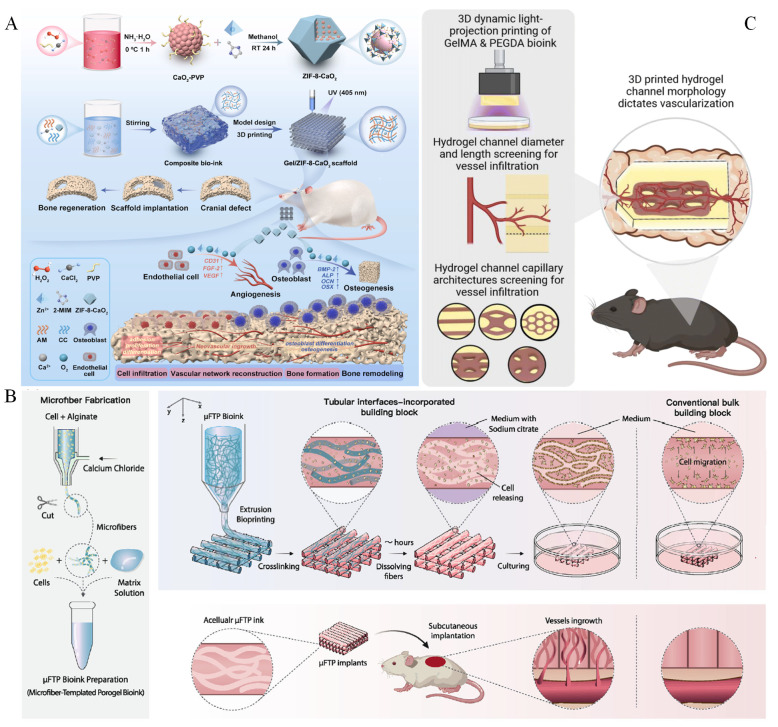
(**A**) Schematic illustration for the preparation of 3D-printed Gel/ZIF-8-CaO2 composite scaffold and the significant effect in augmented vascularized bone regeneration [66] (copyright © 2024, Yang et al. under the terms of the Creative Commons CC BY 4.0). (**B**) Schematic of formulating μFTP bioink for bioprinting applications [67] (adapted with permission from Guo et al. copyright 2025 John Wiley and Sons). (**C**) Schematic diagram of the development and screening for hydrogel channel morphology optimized for enhanced vascular infiltration [68] (copyright © 2025, Fowler et al. under the terms of the Creative Commons CC BY 4.0).

Hydrogel scaffolds provide a hydrated and cell-friendly matrix for tissue repair, but conventional hydrogel scaffolds often have limited ability to guide vascular formation. Dense or poorly interconnected hydrogel networks may restrict oxygen diffusion, cell infiltration, and vessel ingrowth. Therefore, hydrogel scaffolds designed for vascularized regeneration should not only be biocompatible, but also possess interconnected pores, perfusable channels, suitable degradation behavior, and pro-angiogenic microenvironments. In this context, 3D printing provides a powerful strategy for engineering vascularized hydrogel scaffolds because it enables precise control over channel geometry, pore size, interconnectivity, and spatial distribution of oxygen-generating components, cells, or angiogenic signals. For vascularized bone regeneration, 3D printing is particularly valuable because it allows structural customization and biological regulation to be integrated within one scaffold. Yang et al. constructed a self-oxygenating 3D-printed bioactive hydrogel scaffold by integrating oxygen-generating nanoparticles with a hybrid double-network hydrogel (Figure 3A) [66]. This scaffold achieved long-term oxygen generation, structural customization, and excellent mechanical properties, thereby significantly enhancing vascularized bone regeneration without the aid of cells or growth factors. This study suggests that oxygen-supplying hydrogel scaffolds can relieve hypoxia in bone defects and promote vascularized regeneration through a cell-free and growth factor-free strategy. However, oxygen supply alone may not be sufficient to reconstruct organized vascular networks, especially at the microscale. Therefore, recent studies have increasingly focused on engineering vascular structures directly within the building blocks of printed hydrogels. Guo et al. introduced a new type of bioink called microfiber-templated porogel bioink to engineer vasculatures down to the filament level of 3D-bioprinted hydrogels (Figure 3B) [67]. The resulting bioprinted scaffolds significantly promoted blood vessel and native tissue ingrowth in vivo. More importantly, this strategy enabled the formation of tubular bio-interfaces within printed filaments and contributed to in situ endothelialization of microvasculatures, providing a versatile platform for customized vascularized tissue models. In addition to extrusion-based strategies, light-based printing methods also provide powerful tools for designing vascularized hydrogel scaffolds with well-defined architectures. Fowler et al. used digital light processing to 3D bioprint GelMA/Poly(ethylene glycol) diacrylate (PEGDA) hydrogels with different channel architectures and evaluated their effects on tissue infiltration and vascularization in rodent models (Figure 3C) [68]. The GelMA/PEGDA hydrogels showed good mechanical robustness and biocompatibility and supported in vivo vascular infiltration. Notably, channel diameter significantly influenced vascularization, with 1 mm channels yielding the highest infiltration, whereas channel length had minimal impact. Among the five tested architectures, the GEO3 design promoted the greatest vascular ingrowth, demonstrating that channel architecture can be tuned to guide vascular infiltration within hydrogel scaffolds.

Overall, vascularized regeneration requires 3D-printed hydrogel scaffolds that can simultaneously regulate mass transport, oxygen supply, cell infiltration, and vascular organization. For bone regeneration, vascularization is essential for coupling angiogenesis with osteogenesis and preventing hypoxia-induced necrosis in large defects. For broader tissue engineering applications, vascularized hydrogel scaffolds should contain interconnected pores, perfusable channels, endothelializable interfaces, and pro-angiogenic microenvironments. Therefore, future 3D-printed hydrogel scaffolds for vascular regeneration should move beyond simple porous structures toward oxygen-generating, microvascularized, and architecture-guided systems that can support functional vascular network formation and integration with host tissues.

## 6. Vascular Constructs

Promoting vascular regeneration is one of the important functions of hydrogel scaffolds in tissue engineering. In addition to promoting vascular ingrowth within regenerative scaffolds, the rapid development of bioprinting technologies has enabled hydrogel scaffolds to be directly fabricated as vascular constructs [69,70]. In this context, 3D-printed hydrogel vascular constructs can be regarded as artificial blood vessel-like structures, aiming to mimic the geometry, mechanical properties, lumen architecture, and biological functions of native blood vessels. Unlike conventional porous scaffolds that mainly rely on host vessel invasion, directly printed vascular constructs can provide preformed hollow channels for perfusion and cell organization. These constructs can be designed with controllable inner diameter, wall thickness, branching geometry, and multilayered architectures, thereby better reproducing the structural features of natural blood vessels.

**Figure 4:**
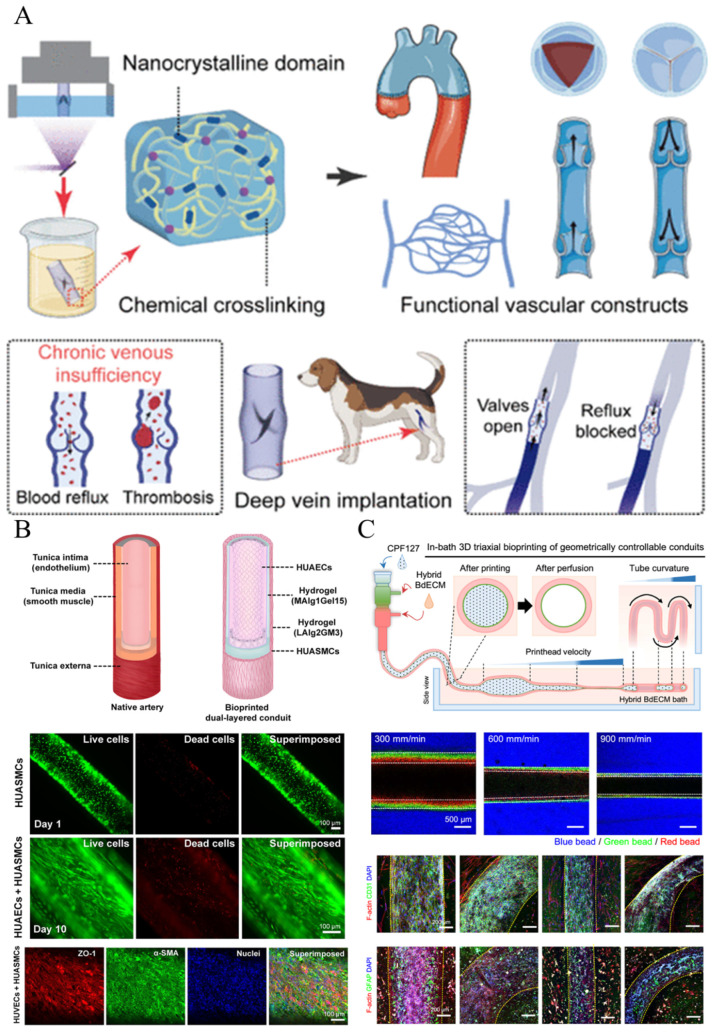
(**A**) Fabrication of hydrogel vascular constructs using high-fidelity digital light processing printing with poly(vinyl alcohol)-based inks [71] (adapted with permission from Ye et al. copyright 2024 American Chemical Society). (**B**) Structural and biological functions of printed veinous conduits [72] (copyright © 2022, Wang et al. under the terms of the Creative Commons CC BY 4.0). (**C**) direct fabrication of multilayered conduits using a triaxial nozzle [73] (copyright © 2023, Park et al. under the terms of the Creative Commons CC BY 4.0).

For large-caliber or implantable vascular constructs, mechanical strength, suture tolerance, swelling resistance, antithrombotic properties, and long-term patency are key requirements. Ye et al. fabricated hydrogel vascular constructs using high-fidelity digital light processing printing with poly(vinyl alcohol)-based inks (Figure 4A) [71]. After printing, the hydrogel vascular constructs were mechanically strengthened by engineering nanocrystalline domains and further modified on the surface to improve their biological performance. The resulting high-precision hydrogel vascular constructs exhibited excellent mechanical stability, suture tolerance, swelling resistance, antithrombotic properties, and long-term patency. This study demonstrates the potential of 3D-printed hydrogel vascular constructs for treating vascular diseases such as chronic venous insufficiency. In addition to photopolymerization-based printing, coaxial bioprinting is also an important strategy for constructing tubular hydrogel vascular structures. Coaxial bioprinting is a specialized extrusion-based printing technique that uses a multi-channel coaxial nozzle to simultaneously extrude multiple bioinks in a concentric manner, thereby forming hollow tubular structures in a single step. This method is particularly suitable for mimicking the multilayered structure of native blood vessels, including the inner endothelial layer, middle smooth muscle layer, and outer adventitial layer, which together maintain normal vascular function. During coaxial printing, the core and shell inks can rapidly undergo ionic or thermal gelation when they contact the corresponding crosslinking agents at the nozzle tip, forming continuous hollow filaments with tunable lumen diameter and wall thickness. This concentric printing strategy enables the one-step fabrication of multilayered perfusable tubular scaffolds and provides an important technical basis for constructing hydrogel-based artificial blood vessels and microvascular networks. Therefore, coaxial printing is especially suitable for fabricating hydrogel vascular constructs with perfusability, multilayered architecture, and potential endothelialization capacity (Figure 4B) [72]. At smaller scales, the application of printed vascular constructs has gradually shifted from mechanical replacement toward the simulation of vascular microphysiology. Coaxial printing has been widely used in organ-on-a-chip systems and in vitro disease models. In these platforms, the focus is no longer limited to obtaining mechanically robust vascular substitutes, but instead extends to modeling endothelial barrier function, inflammation-driven vascular remodeling, flow-related pathological processes, and tumor cell extravasation. Submillimeter-scale microvessels fabricated by coaxial printing can be integrated into microfluidic devices to study endothelial barrier integrity, inflammatory responses, and interactions between tumor cells and vascular interfaces. Recent studies have further shown that stenosis-mimicking vascular structures can be constructed using embedded 3D coaxial bioprinting in combination with mechanically reinforced bioinks and support baths (Figure 4C) [73]. Such systems provide powerful platforms for modeling flow-mediated vascular pathologies in vitro. This strategy can also be extended to more complex vascular microenvironments, such as cerebral vascular models incorporating astrocytes or tumor vascular models for studying the influence of vascular geometry on cancer cell extravasation. These applications indicate that 3D-printed hydrogel vascular constructs can not only be used for vascular graft fabrication but also serve as important platforms for the mechanistic studies of vascular biology. However, although current 3D printing technologies can relatively readily fabricate perfusable channels or hollow tubular structures, the reconstruction of aortic or venous walls remains much more challenging. This is because the aortic wall and venous wall are not simple conduits, but complex tissue structures with distinct structural and mechanical requirements. The aortic wall needs to withstand high pulsatile blood pressure and therefore requires high mechanical strength, elasticity, and compliance, whereas the venous wall is generally thinner and more compliant but requires sufficient anti-collapse ability, hemocompatibility, and long-term patency. Native blood vessels also have complex multilayered architectures, anisotropic mechanical properties, antithrombotic luminal surfaces, and long-term remodeling capacity, all of which are still difficult to fully reproduce using current hydrogel-based printing strategies. Therefore, for implantable vascular grafts intended for aortic or venous wall reconstruction, the design should go beyond the fabrication of simple tubular conduits. Future 3D-printed hydrogel vascular constructs should further integrate structural fidelity, mechanical stability, surface biofunctionality, compliance matching, and vascular cell organization to better mimic the multilayered architecture, mechanical behavior, and biological functions of native blood vessels.

## 7. Neural Regeneration

Neural regeneration is another important application of hydrogel scaffolds in tissue engineering. Unlike tissues with strong regenerative capacity, the nervous system has limited self-repair ability after injury, especially in cases of peripheral nerve defects, spinal cord injury, and traumatic brain injury. Neural repair is challenging because regenerating axons must extend over long distances, maintain directional guidance, reconnect with target tissues, and restore functional neural signaling [74,75]. In addition, the injured neural microenvironment is often accompanied by inflammation, scar formation, cell loss, and insufficient neurotrophic support, all of which hinder effective regeneration [76,77].

**Figure 5:**
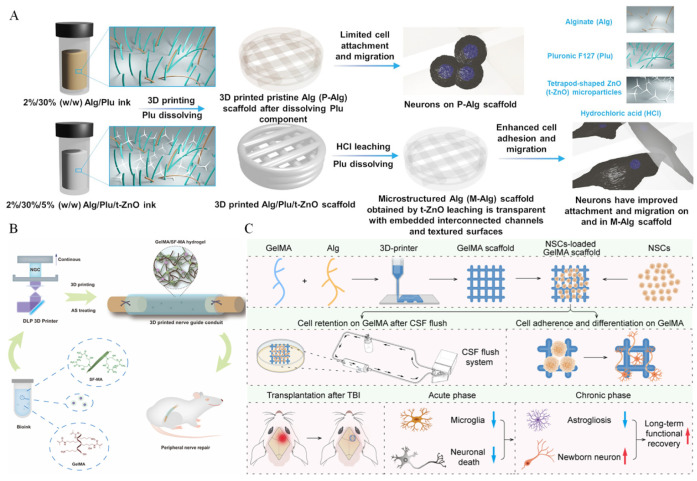
(**A**) 3D-printed alginate scaffolds showing enhanced neuron adhesion and proliferation [78] (copyright © 2025, Li et al. under the terms of the Creative Commons CC BY 4.0). (**B**) 3D-printed elastic GelMA/SF-MA hydrogel conduits with 7,8-DHF prodrug nanoassemblies for peripheral nerve repair [79] (copyright © 2023, Wu et al. under the terms of the Creative Commons CC BY 4.0). (**C**) Schematic illustration of mechanism for printed NSCs-loaded GelMA/Alg scaffold [80] (copyright © 2023, Chen et al. under the terms of the Creative Commons CC BY 4.0).

Hydrogel scaffolds are attractive for neural tissue engineering because their soft and hydrated networks can partially mimic the mechanical properties and extracellular matrix-like environment of neural tissues. For neural regeneration, an ideal hydrogel scaffold should provide a permissive microenvironment for neuronal adhesion, neurite outgrowth, Schwann cell migration, neural stem cell survival, and axonal guidance. In addition, the scaffold should possess appropriate elasticity, interconnected porous structures, directional cues, and controlled delivery capacity for neurotrophic factors or therapeutic molecules. However, conventional bulk hydrogels often lack sufficient topographical guidance and may not effectively support oriented axonal extension or neural network maturation. In this context, 3D printing provides an effective strategy for constructing hydrogel scaffolds with defined microstructures, channels, and spatially organized biochemical cues. Through precise architectural design, 3D-printed hydrogel scaffolds can provide physical guidance for neurite extension and cell migration. For peripheral nerve repair, 3D printing can be used to fabricate nerve guidance conduits with customized geometries, internal channels, and drug-release functions. For central nervous system repair, printed hydrogel scaffolds can serve as carriers for neural stem cells and help improve cell retention, survival, and differentiation in the injured region. For example, Li et al. demonstrated proof-of-concept, bioactive additive-free microstructured alginate scaffolds for neuron culture (Figure 5A) [78]. In this study, tetrapod-shaped ZnO microparticles were introduced into the printing ink as removable structural templates to generate interconnected channels and textured surfaces within the 3D-printed alginate scaffold. After removal of the ZnO templates, the resulting microstructured alginate scaffold showed significantly improved neuronal adhesion and growth compared with pristine alginate scaffolds. Neurons cultured on these scaffolds exhibited extensive neurite outgrowth and spontaneous neural activity, indicating the maturation of neuronal networks. This study suggests that microstructural design alone, even without additional bioactive additives, can improve the neuron affinity of alginate-based hydrogel scaffolds and support neural network formation. In another study, Wu et al. fabricated an elastic hydrogel conduit encapsulating prodrug nanoassemblies using a continuous 3D printing technique for peripheral nerve regeneration (Figure 5B) [79]. The bioactive hydrogel conduit was composed of gelatin methacryloyl and silk fibroin glycidyl methacrylate, which provided favorable properties for Schwann cell adhesion, proliferation, and migration. Meanwhile, 7,8-dihydroxyflavone prodrug nanoassemblies with high drug-loading capacity were incorporated into the hydrogel conduit. The drug-loaded conduit enabled sustained release of 7,8-dihydroxyflavone, thereby facilitating neurite elongation and promoting nerve regeneration. This work highlights the value of combining 3D-printed hydrogel conduits with sustained neuroactive molecule delivery for peripheral nerve repair. Hydrogel scaffolds can also serve as carriers for neural stem cell transplantation in central nervous system injury. In traumatic brain injury, cerebrospinal fluid flow may contribute to cell loss after neural stem cell transplantation, thereby reducing therapeutic efficacy. Hydrogel scaffolds offer a potential solution by providing a supportive matrix that improves cell retention and protects transplanted cells from mechanical loss. In one study, several hydrogel scaffolds were evaluated, and the gelatin methacryloyl/sodium alginate hydrogel (GelMA/Alg) scaffold showed the best performance in supporting neural stem cell adhesion, growth, and differentiation (Figure 5C) [80]. Moreover, pre-differentiated neural stem cells loaded on the GelMA/Alg hydrogel and cultured in neuronal differentiation medium for seven days exhibited the highest cell retention rate after cerebrospinal fluid impact. This suggests that hydrogel scaffolds can improve stem cell delivery and retention in neural repair, particularly under mechanically dynamic conditions such as traumatic brain injury.

Overall, neural regeneration requires hydrogel scaffolds that provide both structural guidance and biological support. For peripheral nerve repair, 3D-printed hydrogel conduits should guide axonal extension, support Schwann cell migration, and deliver neuroactive molecules in a sustained manner. For neuronal culture and neural organoid-related studies, microstructured hydrogel scaffolds can promote neuronal adhesion, neurite outgrowth, and network maturation. For central nervous system injury, hydrogel scaffolds can improve neural stem cell retention, survival, and differentiation. Therefore, future 3D-printed hydrogel scaffolds for neural regeneration should integrate soft mechanical properties, oriented microstructures, interconnected channels, controlled release functions, and cell-supportive microenvironments to better promote functional neural repair.

## 8. Wound Healing

Wound healing is another important application of 3D-printed hydrogel scaffolds in tissue engineering. Skin is the largest barrier tissue of the human body and plays essential roles in preventing water loss, pathogen invasion, and external mechanical damage [81,82]. Once full-thickness skin defects or chronic wounds occur, the repair process becomes more complex because it involves hemostasis, inflammation regulation, fibroblast migration, angiogenesis, extracellular matrix remodeling, and re-epithelialization. In particular, chronic wounds such as diabetic wounds often suffer from persistent inflammation, impaired angiogenesis, oxidative stress, bacterial infection, and delayed tissue remodeling, making spontaneous healing difficult [58,83].

Hydrogel scaffolds are highly suitable for wound repair because they provide a moist and biocompatible microenvironment that resembles the extracellular matrix of skin tissue. They can absorb wound exudate, maintain local hydration, support cell adhesion and migration, and serve as carriers for cells, antibacterial agents, growth factors, exosomes, or other bioactive molecules. However, conventional hydrogel dressings often lack structural complexity and may not fully reproduce the layered architecture and multifunctional properties of native skin. For full-thickness skin repair, an ideal hydrogel scaffold should not only cover the wound surface, but also promote hemostasis, reduce inflammation, prevent infection, support angiogenesis, and guide epidermal and dermal regeneration.

**Figure 6:**
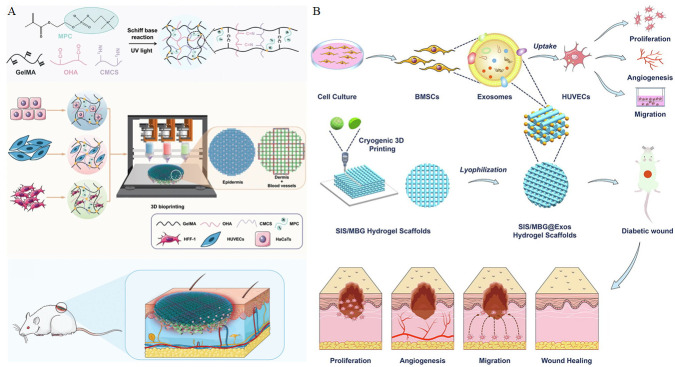
(**A**) 3D Bioprinting of Double-Layer Conductive Skin for Wound Healing [84] (adapted with permission from Wang et al. copyright 2025 John Wiley and Sons). (**B**) 3D-printed hydrogel scaffolds loading exosomes accelerate diabetic wound healing [85] (copyright © 2021, Hu et al. under the terms of the Creative Commons CC BY 4.0).

In this context, 3D bioprinting provides an effective strategy for constructing skin-like hydrogel scaffolds with customized geometry, multilayer architecture, and spatially organized cells or bioactive components. Compared with conventional wound dressings, 3D-printed hydrogel scaffolds can better match irregular wound shapes and reproduce the layered structure of skin. In addition, 3D printing allows the incorporation of multiple cell types and functional components into different regions of the scaffold, thereby providing a more biomimetic microenvironment for skin regeneration. For example, Wang et al. reported the 3D bioprinting-assisted fabrication of a double-layer ionic conductive skin scaffold using a newly designed ionic conductive biomimetic bioink for full-thickness skin defect repair. The bioink was composed of gelatin methacrylate, oxidized hyaluronic acid, carboxymethyl chitosan, and 2-methacryloyloxyethyl phosphorylcholine (Figure 6A) [84]. The combination of a rigid GelMA network and a dynamic oxidized hyaluronic acid-carboxymethyl chitosan (OHA-CMCS) network endowed the bioink with reversible thixotropy, good printability, high shape fidelity, and favorable cell activity during one-step bioprinting. Moreover, the incorporation of zwitterionic MPC provided electrical signaling properties similar to those of natural skin tissue. By integrating human foreskin fibroblasts, human umbilical vein endothelial cells, and human immortalized keratinocytes, the researchers constructed a double-layer conductive skin scaffold consisting of an epidermal layer and a vascularized dermal layer. This study demonstrates that 3D bioprinting can integrate structural mimicry, cellular organization, and bioelectrical functionality into hydrogel skin scaffolds. Besides full-thickness skin reconstruction, 3D-printed hydrogel scaffolds are also promising for chronic wound healing, especially diabetic wounds. Hu et al. used extrusion-based cryogenic 3D printing to construct a 3D scaffold dressing composed of decellularized small intestinal submucosa, mesoporous bioactive glass, and exosomes (Figure 6B) [85]. The resulting small intestinal submucosa combined with mesoporous bioactive glass and exosomes (SIS/MBG@Exos) scaffold allowed sustained release of bioactive exosomes and possessed suitable porosity, good biocompatibility, and hemostatic ability. In vitro results showed that the scaffold promoted the proliferation, migration, and angiogenic activity of human umbilical vein endothelial cells. In diabetic wound models, the SIS/MBG@Exos hydrogel scaffold accelerated wound healing by increasing local blood flow and stimulating angiogenesis. This work suggests that combining 3D-printed porous hydrogel scaffolds with extracellular matrix components, bioactive inorganic materials, and exosomes can provide a multifunctional strategy for chronic wound repair.

Overall, wound healing requires hydrogel scaffolds with multiple coordinated functions, including wound coverage, moisture retention, hemostasis, antibacterial activity, inflammation regulation, angiogenesis, and tissue remodeling. For full-thickness skin defects, 3D-printed hydrogel scaffolds should mimic the layered architecture of skin and support the coordinated regeneration of epidermal and dermal tissues. For chronic wounds, especially diabetic wounds, scaffolds should further address impaired vascularization, persistent inflammation, and delayed tissue repair. Therefore, future 3D-printed hydrogel scaffolds for wound healing should move beyond simple wound dressings toward multifunctional, biomimetic, and cell-instructive skin substitutes that integrate structural design, bioactive delivery, electrical signaling, and vascularization-promoting functions.

## 9. Discussion

**Figure 7:**
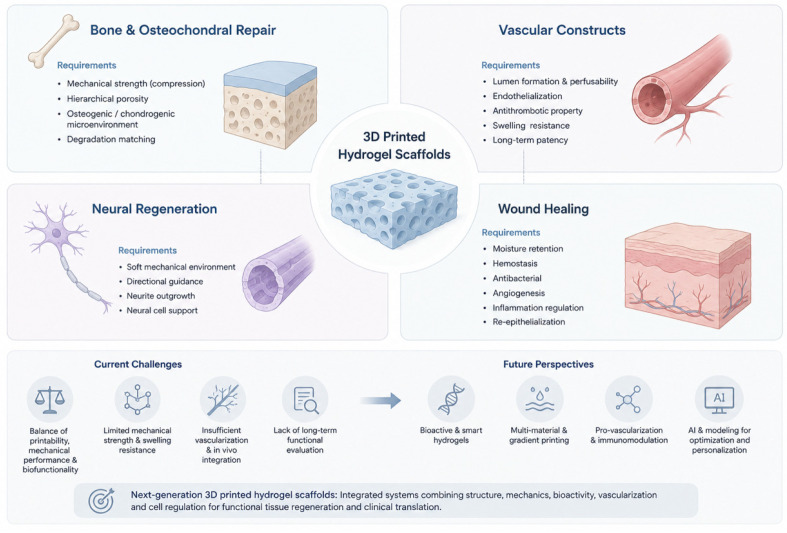
Applications, limitations, and future perspectives of 3D-printed hydrogel scaffolds.

3D-printed hydrogel scaffolds have shown broad potential in multiple tissue engineering applications, including bone and osteochondral repair, vascular regeneration, neural regeneration, and wound healing. These studies demonstrate that hydrogel scaffolds are no longer simple passive fillers, but functional three-dimensional platforms that provide structural support, regulate cellular behavior, deliver bioactive cues, and guide tissue regeneration (Figure 7). However, different tissues require distinct scaffold properties. Bone and osteochondral repair require compressive resistance, hierarchical porosity, mechanical gradients, and osteogenic or chondrogenic microenvironments. Vascular constructs require lumen formation, perfusability, endothelialization, antithrombotic properties, swelling resistance, and long-term patency. Neural regeneration requires soft mechanical properties, directional guidance, neurite outgrowth, and neural cell support. Wound healing requires moisture retention, hemostasis, antibacterial activity, angiogenesis, inflammation regulation, and re-epithelialization. Therefore, the design of 3D-printed hydrogel scaffolds should not rely on a universal strategy but should instead be tailored to tissue-specific structural and functional requirements.

Although 3D printing greatly improves the structural controllability of hydrogel scaffolds, balancing printability, mechanical performance, and biofunctionality remains a major challenge. Hydrogel inks with high viscosity or rapid crosslinking usually provide better shape fidelity and structural stability, but they may restrict cell spreading, migration, nutrient diffusion, and matrix remodeling. In contrast, hydrogels with good cell compatibility are often too soft to maintain complex three-dimensional structures. This issue is particularly important for bone, osteochondral, and vascular graft applications, where pure hydrogels often fail to provide sufficient compressive strength, tensile resistance, suture tolerance, and long-term stability. Strategies such as double-network hydrogels, nanocomposite reinforcement, mineralization, natural–synthetic polymer hybrids, and multi-material printing can improve scaffold performance, but excessive reinforcement may compromise cell viability, degradation matching, or tissue integration. Vascularization, functional maturation, and in vivo integration remain key challenges limiting the broader application of 3D-printed hydrogel scaffolds. For large-volume tissue engineering, insufficient vascularization can lead to hypoxia, nutrient deprivation, and tissue necrosis in the central region of scaffolds. Although 3D printing can generate pores, channels, and vascular-like networks, constructing functional vascular systems that are perfusable, endothelialized, and integrated with host vessels remains difficult. Moreover, many current studies still focus on cell viability, proliferation, or early differentiation markers, whereas tissue-specific functional outcomes are more important. For example, bone repair should evaluate mineralization and mechanical integration, cartilage repair should assess type II collagen and glycosaminoglycan deposition, vascular constructs should focus on long-term patency and endothelial barrier function, neural regeneration should evaluate neurite extension, neural activity, and functional recovery, and wound healing should assess re-epithelialization, vascularization, and scar control. In the future, the development of 3D-printed hydrogel scaffolds should move beyond printability and shape fidelity toward tissue-specific biofunctionality and clinical translation. From a translational perspective, implantable hydrogel scaffolds still face several challenges before routine clinical application, including reproducible manufacturing, sterilization, storage stability, surgical handling, mechanical reliability, degradation control, immune compatibility, and long-term safety. Nevertheless, some biomaterial-based scaffold products have already entered clinical use or commercialization, providing useful references for the translation of 3D-printed hydrogel scaffolds. For example, chitosan-based nerve guidance conduits, such as Reaxon^®^ Nerve Guide, have been clinically investigated and used for peripheral nerve repair, demonstrating the feasibility of using biodegradable polymer conduits as implantable regenerative scaffolds. These clinical and commercial examples suggest that the translation of scaffold implants is possible, but successful clinical application requires not only favorable biological performance, but also reliable manufacturing, appropriate mechanical properties, regulatory compliance, and validated long-term functional outcomes.

In addition, artificial intelligence and computational modeling are expected to play an increasingly important role in the rational design of 3D-printed hydrogel scaffolds. At the material level, machine learning can be used to analyze the relationships among hydrogel composition, polymer concentration, crosslinking density, rheological behavior, printability, degradation rate, and mechanical properties, thereby accelerating the screening of suitable bioink formulations. At the printing-process level, machine learning can help optimize key printing parameters, such as extrusion pressure, nozzle diameter, printing speed, layer height, temperature, light intensity, exposure time, and crosslinking conditions. These parameters directly affect filament uniformity, shape fidelity, pore accuracy, interlayer bonding, cell damage, and post-printing scaffold stability. At the structural level, computational modeling and optimization algorithms can help design scaffold architectures with appropriate pore size, porosity, interconnectivity, channel geometry, and mechanical anisotropy according to the requirements of different tissues. For example, finite element analysis can predict stress distribution and deformation behavior within printed scaffolds, while machine learning models can further correlate material properties, printing parameters, and structural features with biological outcomes such as cell viability, proliferation, differentiation, vascularization, and extracellular matrix deposition. Moreover, image-based modeling using CT, MRI, or 3D scanning data can support the design of patient-specific scaffolds that match irregular defect geometries. By integrating experimental datasets, computational simulation, and machine learning-guided optimization, future scaffold design may gradually shift from trial-and-error fabrication toward data-driven and tissue-specific precision manufacturing. Overall, next-generation 3D-printed hydrogel scaffolds should be designed as integrated systems that combine suitable materials, precise architectures, controlled bioactive delivery, mechanical adaptation, vascularization, and tissue-specific cellular regulation, thereby promoting their translation from laboratory constructs to functional regenerative platforms with clinical potential.

## 10. Conclusions

3D-printed hydrogel scaffolds have emerged as versatile platforms for tissue engineering by combining the ECM-like properties of hydrogels with the structural controllability of 3D printing. As discussed in this review, their applications in bone and osteochondral repair, vascular regeneration, neural regeneration, and wound healing demonstrate that different tissues require distinct scaffold designs, including specific mechanical properties, architectures, degradation behaviors, and bioactive cues. Therefore, the development of hydrogel scaffolds should move beyond simple printability and shape fidelity toward tissue-specific biofunctionality. Despite significant progress, challenges remain in balancing printability, mechanical strength, cell compatibility, vascularization, functional maturation, and clinical translation. Future advances in smart hydrogels, multi-material printing, bioactive delivery, vascularized constructs, and patient-specific design may further improve the regenerative performance of 3D-printed hydrogel scaffolds. Overall, next-generation 3D-printed hydrogel scaffolds are expected to serve as integrated biomimetic systems that guide functional tissue regeneration and promote their translation toward clinical applications.

## Figures and Tables

**Table 1:** Effects of 3D printing methods on scaffold performance.

Printing Methods	Key Ink Requirements	Examples of Polymers	Advantages	Disadvantages	References
Extrusion-based Printing	Tunable rheology, shear-thinning, balanced viscosity, cell encapsulation, mechanical support	Gelatin, Alginate, Collagen, PEG derivatives, PVA	No need for complex bioink modification, many natural extruded bioinks are suitable for cell encapsulation, survival, and growth, good proliferation	Shear stress-induced reduction in cell viability, weak mechanical properties, lower resolution than photopolymerization	[25,26]
Photopolymerization-based Printing (e.g., SLA, Volume Printing)	Photocrosslinkable, C=C functional groups, appropriate photoinitiators	GelMA, PEGDA, modified natural polymers	High resolution, fine features, overhanging structures, rapid bulk fabrication, good proliferation	Photoinitiator cytotoxicity induced reduction in cell viability, requires light optimization	[41,42]

**Table 2:** Effects of bioinks on scaffold performance.

Polymer Type	Examples	Advantages	Disadvantages	References
Natural Polymers	Gelatin, alginate, collagen	Biocompatible, bioactive motifs, promotes cell adhesion, proliferation, differentiation	Rapid degradation, time-consuming rheology optimization process, risk of nozzle clogging, unsuitable for photopolymerization-based printing	[48,49]
Synthetic Polymers	PEG derivatives, PVA, Polycarbonates	Tunable mechanical properties, stable quality, chemical functionalization	Photoinitiator cytotoxicity, dependence on strict photopolymerization control, absence of cell-adhesive binding sites	[50,51]

## Data Availability

Not applicable. As this is a review article, the data supporting this article can be found in the original articles discussing each topic.
